# The Oculo-cardiac Reflex

**Published:** 1915-11

**Authors:** 


**The Oculo-cardiac Reflex.** This phenomenon, which was first described in 1908 by Aschner, and to which considerable attention has recently been paid, consists in a change in the pulse rate, usually a slowing, and sometimes in a change in the pulse rythm, following compression of the ocular bulb. The reflex occurs in normal individuals, its path consisting of the fifth nerve, the medulla, and the vagus or sympathetic nerve. Persons showing the usual type of this reflex, in which the vagus forms part of the arc, are designated as vago-tonics; persons in whom the sympathetic is part of the reflex arc react to ocular compression by means of a very slight slowing or an actual increase of the pulse; these individuals are known as sympathicotonics.

Two important clinical studies of the oculo-cardiac reflex have recently appeared. The first of these, by Noel Orlandi, is contributed to Riforma Medica, February 27 and March 6 and 13, 1915. He finds that compression of the ocular bulb causes a slowing of the pulse in normal persons to the extent of four to twelve pulsations a minute. The same effect is observed whether the two bulbs are compressed simultaneously or alternately. If compression is repeated three or four times at intervals of a few minutes the reflex disappears. It may show considerable variation in the same individual; on one day light compression of the bulb may cause a reduction of twelve or fourteen in the pulse rate, whereas on the following day similar compression may cause a reduction of only four. In no normal subject has Orlandi ever observed an inversion of the reflex. In two instances, upon the repetition of the test following the production of bradycardia he obtained a transient acceleration of the pulse. Age has an important influence upon the phenomenon. Infants react with a more pronounced bradycardia than do adults; in women the reflex is more marked than in men. On the basis of his observations Orlandi concludes that the oculo-cardiac reflex is a normal phenomenon dependent upon the integrity of the vagus path; apparently, however, the reflex arc is frequently exhausted, for in certain instances the repetition of the stimulus causes the nerve impulse to be propagated along the sympathetic, in which case tachycardia results.

The oculo-cardiac is abolished in chronic aortitis. It is absent in tabes dorsalis with a constancy comparable with the presence of the Argyll-Robertson pupil. The reflex is exaggerated in the vagotonic syndrome and inverted in the sympathicotonic syndrone. It is a useful diagnostic aid in cases of bradycardia and in certain cases of changed conductivity in the atrioventricular path, by showing the integrity or impairment of the extracardiac innervation In other words, the oculo-cardiac reflex is an index of the functional efficiency of the vagus. But before attributing any diagnostic value to this reflex one must bear in mind the wide limits of its variability under normal conditions, and the fact that the reflex is easily exhausted by repetition.

E. B. Gunson (British Journal of Children's Diseases, April, 1915), has studied the oculo-cardiac reflex with the aid of the polygraph in cases of diphtheria and scarlet fever occurring in children under twelve years of age. He notes that the positive vagal effect or slowing of the pulse is an indication of the normal state of the nervous mechanism of the heart, and may be obtained even in the presence of a myocardial degeneration. The reflex is positive in about 92 per cent. of children convalescent from diphtheria and scarlet fever. The negative reflex, that is, one in which there is no slowing or an actual quickening of the pulse, occurs in all cases of so-called "cardiac paralysis" that terminate fatally; in cases that recover the reflex becomes positive when the heart returns to the normal state.

The study of the positive oculo-cardiac reflex in cases of scarlet fever and diphtheria with the aid of the polygraph shows a slowing of the whole pulse, with stoppage of the heart in some cases for as long as four seconds; a production of premature contractions; a reduction of the a—c interval; a production of c+a beats; and in diphtheria cases only, a complete dissociation of auricles and ventricles. Gunson concludes that the oculo-cardiac reflex is of diagnostic value in confirming the neural origin of the great majority of the post-febrile brady-cardias and in differentiating them from cases of auriculo-ventricular heart block.—N. Y. Medical Record. (This phenomenon is fully discussed in Aschner's work on Diseases of the Digestive Organs).

